# Assessment of the Cardioankle Vascular Index in Pregnant Women Complicated with Hypertensive Disorders

**DOI:** 10.5402/2011/919816

**Published:** 2011-05-25

**Authors:** Atsushi Yoshida, Takashi Sugiyama, Norimasa Sagawa

**Affiliations:** ^1^Department of Obstetrics and Gynecology, Nishisaitama-Chuo National Hospital, 2-1671 Wakasa, Tokorozawa, Saitama 359-1151, Japan; ^2^Department of Obstetrics and Gynecology, Mie University School of Medicine, 2-174 Edobashi, Tsu, Mie 514-8507, Japan

## Abstract

It was previously reported that the brachial-ankle pulse wave velocity (baPWV) is elevated in preeclamptic women. However, baPWV is strongly affected by blood pressure. Recently, a new index of vascular stiffness, the cardioankle vascular index (CAVI), was developed. CAVI is thought to be an index independent of blood pressure. We assessed CAVI in normotensive and hypertensive pregnant women. We studied a total of 109 Japanese women consisting of 23 nonpregnant healthy women (group A), 45 normotensive pregnant women (group B), 28 pregnant women complicated with established preeclampsia (group C), and 13 pregnant women with chronic hypertension (group D). The subject remained supine while the blood pressure, baPWV, and CAVI were recorded. No significant difference in baPWV was present between groups C and D, but the difference in CAVI was significantly high in group D. We believe that we can distinguish the vessel structural change between chronic hypertension and preeclampsia through simultaneous baPWV and CAVI measurements.

## 1. Introduction

For early detection of arteriosclerotic disease, several parameters for assessing vascular nature have been used. The pulse wave velocity (PWV) is one of these parameters and has been widely used mainly in the field of internal medicine, especially in the prediction of cardiovascular events [1]. The original PWV was measured as the velocity between the carotid and femoral artery. However, carotid-femoral PWV requires somewhat complicated technique to obtain an accurate waveform. Brachial-ankle PWV has been developed, which is reported to be correlated with carotid-femoral PWV and can be measured with a simpler technique. It was also reported that PWV is elevated in preeclamptic women [2]. However, it was pointed out that both carotid-femoral PWV and baPWV were strongly affected by the changes in blood pressure during measurements. For the purpose of using PWV as an indicator of vascular vessel wall stiffness, it is necessary to eliminate the effect of blood pressure change from PWV.

Recently, a new index of vascular stiffness, the cardio ankle vascular index (CAVI), was developed. Considered to be an index independent of blood pressure, CAVI is a parameter of adjusted PWV for blood pressure, based on a stiffness parameter *β* [3] using Bramwell-Hill's formula [4]. CAVI is calculated as follows:

(1) $$\text{CAVI}=a\left(\frac{2\rho }{\Delta P}\right)\times \ln\;\left(\frac{\text{Ps}}{\text{Pd}}\right)\times {\text{PWV}}^{2}+b,$$

where Ps indicates systolic pressure in brachial artery; Pd, diastolic pressure in brachial artery; *ρ*, density of blood; *a*, *b*, correction coefficient.

The usefulness of CAVI in the management of patients with hypertensive, diabetic or renal disorders was previously reported in [5–8], but we found no reports on CAVI in pregnancy. In our current study, we assessed CAVI in normotensive and hypertensive pregnant women. We believe that this is the world's first report on CAVI in pregnancy.

## 2. Subjects and Methods

### 2.1. Study Population

The subjects of this study were 109 Japanese women consisting of 23 nonpregnant healthy women (group A), 45 normotensive pregnant women (group B), 28 pregnant women complicated with established preeclampsia (group C), and 13 pregnant women with chronic hypertension (group D). Eighty-six pregnant women (groups B, C, and D) were patients who visited Mie University Hospital. Twenty-three nonpregnant women (group A) were volunteers including nurses and midwives in Mie University Hospital. All the data in this study were obtained from January 2005 to March 2007. The diagnosis of preeclampsia was made according to the criteria of the International Society for the Study of Hypertension in Pregnancy [9], which includes a blood pressure >140/90 mmHg and >300 mg of protein in a 24-hour urine collection. Chronic hypertension was defined as hypertension diagnosed prior to conception or within the first 20 weeks of pregnancy. The women in group D did not have superimposed preeclampsia. In some cases, the blood pressure recorded during the CAVI examination was lower than the criteria value. The women in group C were normotensive in the first trimester of pregnancy and the blood pressure returned to normal by 12 weeks postpartum. No significant differences were noted in the gestational week and maternal age among the four groups. All the subjects in group A were uncomplicated and uneventful throughout the course of pregnancy and puerperium.

### 2.2. Cardioankle Vascular Index

The CAVI is based on the stiffness parameter (*β*), which expressed blood pressure independent vascular stiffness. Stiffness parameter (*β*) is calculated as follows:

(A) $$\beta =\left[\;\ln\;\left(\frac{\text{Ps}}{\text{Pd}}\right)\;\right]\cdot \left(\frac{D}{\Delta D}\right).$$

ln indicates natural log; Ps, systolic pressure; Pd, diastolic pressure; *D*, vascular diameter; Δ*D*, change of vascular diameter.

The relationship between volume elastic modulus and PWV is expressed by Bramwell-Hill's formula, and the relationship between vessel volume and diameter is given when a cylindrical model is used:

(B) $$\begin{array}{c} Z^{2}=\left(\frac{\Delta P}{\rho }\right)\cdot \left(\frac{V}{\Delta V}\right)\;\left(\text{Bramwell-}{\text{Hill}}^{\prime }\text{s}\;\text{formula}\right), \end{array}$$

(C) $$\begin{array}{c} \frac{V}{\Delta V}=\frac{D}{2\Delta D}. \end{array}$$

*Z* indicates cardioankle PWV (m/sec); Δ*P*, pulse puressure; *ρ*, blood density (1.03 × 10^3^ kg/m^3^); *V*, volume of blood vessel; Δ*V*, change of *V*.

The previous 3 formulas (A, B, and C) give the following equation:

(2) $$\beta =\left(\frac{2\rho }{\Delta P}\right)\cdot \left[\mathrm{In}\;\left(\frac{\text{Ps}}{\text{Pd}}\right)\right]\cdot Z^{2}.$$

Thus, the stiffness parameter (*β*) can be easily calculated by measures of BP and cardioankle PWV. The new index CAVI is expressed as follows:

(3) CAVI=aβ+b.

*a* and *b* indicate the constants to convert *β* to a value that is approximate to conventional aortic PWV for each patient compatibility.

Thus CVI is calculated in the following formula.

(4) $$\text{CAVI}=a\left(\frac{2\rho }{\Delta P}\right)\times \ln\;\left(\frac{\text{Ps}}{\text{Pd}}\right)\times {\text{PWV}}^{2}+b.$$

CAVI has a compatibility with conventional PWV. CAVI is easily measured using Vasera VS-1000 (Fukuda Denshi, Tokyo, Japan), which is now becoming popular. The CAVI is automatically calculated by the instrument and reflects the stiffness parameter (*β*). The CAVI has the great merit that it is not influenced by BP, whereas conventional PWV is strongly influenced by BP. However, CAVI might have an error because of the variety of blood density in each case, where in the formula for calculation of CAVI, a standard value of blood density was used.

### 2.3. Measurement and Statistical Analysis

**Figure 1:**
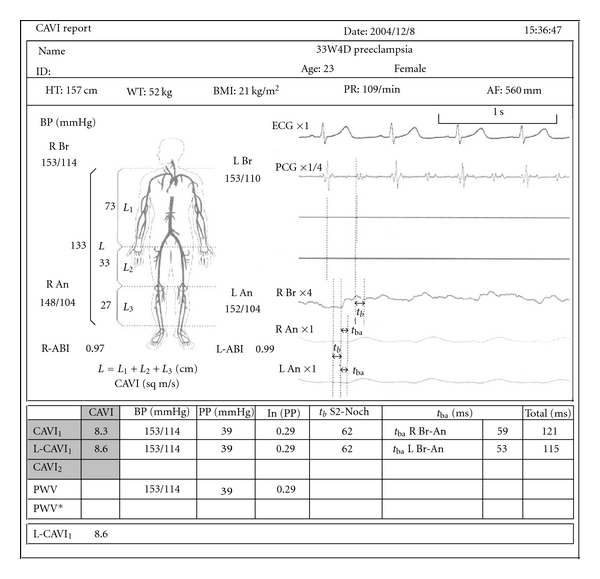
An example of measurement result report in preeclamptic women. In this case, remarkable elevation of baPWV was noted (baPWV = 1812) but CAVI was not markedly elevated (CAVI = 8.3). Actual report is written in Japanese.

The subject was placed in the supine position and blood pressure: baPWV and CAVI were recorded using a vascular screening system VaSera VS-1000 (Fukuda Denshi, Tokyo, Japan). In all cases, no symptomatic adverse effects were noted by supine position.  Figure 1 shows an example of measurement result report in a preeclamptic woman.

Mann-Whitney's *U*-test was used for statistical analysis of comparing values between the two groups. Probability (*P*) values less than  .05 were considered as significant.

## 3. Results

Successful recordings were obtained from all women. The demographic characteristics of the study subjects were shown in Table 1. The values of blood pressure shown in Table 1 were those recorded during the CAVI examination and in some cases the values were lower than the criteria values. Mean gestational age of group B was lower than those in groups C and D, but no significant differences were noted among groups A, B, C, and D in the maternal age, and no significant differences were noted among groups B, C, and D in the gestational age.

**Figure 2:**
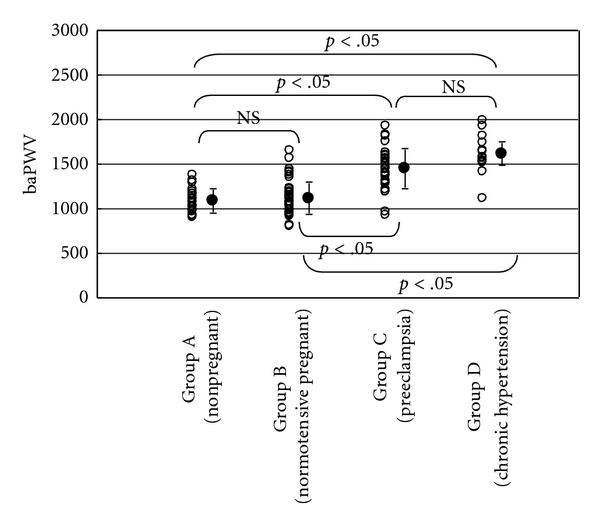
baPWV in each group. NS: not significant.

**Figure 3:**
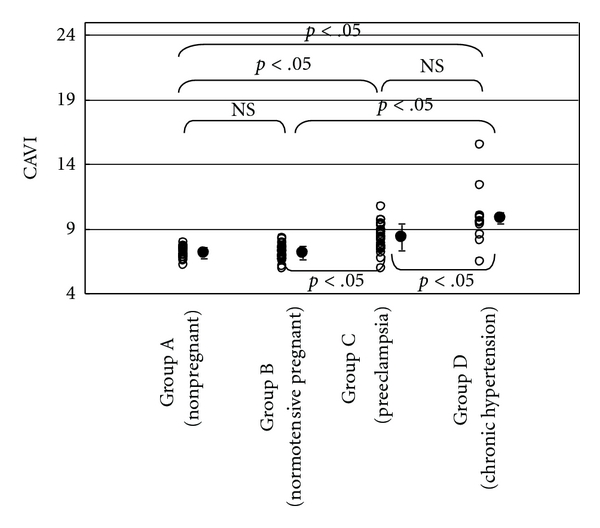
CAVI in each group. NS: not significant.

Significant differences were noted between groups A and C, A and D, B and C, B and D in systolic, diastolic and mean blood pressures. As shown in Figure 2, baPWVs in groups A, B, C, and D were 1087 ± 132 cm/sec, 1119 ± 183 cm/sec, 1446 ± 224 cm/sec, and 1614 ± 225 cm/sec, respectively. Significant differences were noted between groups A and C, A and D, B and C, B and D in baPWV. No significant difference was noted between groups C and D in baPWV. CAVI in each group was shown in Figure 3. CAVI in groups A, B, C, and D were 7.1 ± 0.4, 7.1 ± 0.5, 8.4 ± 1.0, and 9.8 ± 1.8, respectively. Similar to baPWV, significant differences were noted between groups A and C, A and D, B and C, B and D in CAVI. Furthermore, CAVI in group D was significantly higher than that in group C.

## 4. Discussion

To assess the vascular stiffness, several parameters have been used such as carotid-femoral PWV, baPWV, SP*β*, and augmentation index (AI). Carotid-femoral PWV is affected by blood pressure and can be adjusted using a compensating nomogram for blood pressure. However, carotid-femoral PWV requires somewhat complicated technique to obtain an accurate waveform. It is also time-consuming, and it takes approximately 15 to 20 minutes for one measurement. baPWV has been developed, which can be assessed with a simpler technique in a short time using a measurement system but it is strongly affected by the changes in blood pressure during measurement. AI is strongly influenced by heart rate, and it is necessary to correct AI by heart rate for the comparison among subjects. SP*β* is a parameter which is independent of blood pressure but requires a long time for the measurement, and the measuring equipment for SP*β* (sonographic vessel tracking system) is expensive. Moreover, SP*β* is a parameter of the local vessel (usually the abdominal aorta or common carotid artery is used for the measurement) and not a systemic vessel parameter.

CAVI is thought to be an index which eliminates these defects in the conventional vascular parameters. It was previously reported that CAVI is independent of blood pressure [10]. The measuring technique of CAVI is easy and CAVI system is not so expensive. It is a noninvasive method and the repeated measurement during pregnancy is possible. In our current study, we found no complications in the subjects related to CAVI measurements, including pregnant women themselves.

In preeclamptic women, no atherosclerotic change of the vessel wall was produced, which is different from the vessel wall in chronic hypertensive women. In our current study, both in preeclamptic and chronic hypertensive groups, baPWV was elevated and no significant difference was noted between the two groups. On the other hand, CAVI was also elevated both in preeclamptic and chronic hypertensive groups, but the elevation was milder in the former group. Significant difference was noted between the two groups.

In chronic hypertensive women, blood pressure was elevated and (at least partially) structural change in the vessel wall may have been produced, resulting in both baPWV and CAVI elevations. In preeclamptic women, blood pressure was elevated and thus baPWV was elevated. In preeclamptic women, lesser vessel wall structural change was produced, and lesser elevation of CAVI was seen, which is lesser influenced by blood pressure elevation than baPWV. From our results, we believe that we can distinguish the vessel structural change between chronic hypertension and preeclampsia through baPWV and simultaneous CAVI measurements.

However, if CAVI could be completely independent of blood pressure and could completely reflect the atherosclerotic change of the vessel wall, it might be difficult to explain the reason of significant elevation of CAVI in preeclampsia from normotensive pregnancy. Brodszki and colleagues reported that there were no differences in vessel wall stiffness between women with preeclampsia and healthy controls, through the assessment of common carotid artery, abdominal aorta, and popliteal artery by using ultrasonic echo-tracking system [11]. On the other hand, Ong and colleagues reported that structural change was observed in myometrial radial arteries in women with preeclampsia [12]. Thus, we hypothesize that CAVI might reflect the minimal change of the vessel wall, which caused the elevation of CAVI in preeclamptic women. Further work is in progress to evaluate the usefulness of CAVI in the management of preeclampsia.

## 5. Conclusion

Recently, CAVI, a new index of vascular stiffness, was developed, which is thought to be an index independent of blood pressure. We assessed CAVI in normotensive and hypertensive pregnant women as the world's first report on CAVI in pregnancy. We believe that we can distinguish the vessel structural change between chronic hypertension and preeclampsia through simultaneous baPWV and CAVI measurements.

## Figures and Tables

**Table 1:** Demographic characteristics of the study subjects.

	Group A	Group B	Group C	Group D
	Normal healthy	Normotensive pregnant	Preeclampsia	Chronic hypertension
Number of cases	23	45	28	13
Age (years old)	28.3 ± 7.4	30.3 ± 4.9	31.7 ± 6.0	35.8 ± 3.8
Gestational age (weeks)	—	30.4 ± 8.7	33.5 ± 4.8	33.6 ± 3.5
Arterial blood pressure				
Systolic (mmHg)	113 ± 8	111 ± 12	145 ± 17	151 ± 14
Diastolic (mmHg)	70 ± 7	68 ± 9	91 ± 18	96 ± 14
